# Recent Advances in Dual PI3K/mTOR Inhibitors for Tumour Treatment

**DOI:** 10.3389/fphar.2022.875372

**Published:** 2022-05-09

**Authors:** Xianbo Wu, Yihua Xu, Qi Liang, Xinwei Yang, Jianli Huang, Jie Wang, Hong Zhang, Jianyou Shi

**Affiliations:** ^1^ School of Sports Medicine and Health, Chengdu Sport University, Chengdu, China; ^2^ School of Basic Medical Science, Chengdu University of Traditional Chinese Medicine, Chengdu, China; ^3^ College of Medicine, Southwest Jiaotong University, Chengdu, China; ^4^ First Clinical College of Medicine, Guizhou University of Traditional Chinese Medicine, Guiyang, China; ^5^ Hospital of Chengdu University of Traditional Chinese Medicine, Chengdu, China; ^6^ Personalized Drug Therapy Key Laboratory of Sichuan Province, Sichuan Academy of Medical Science and Sichuan Provincial People’s Hospital, School of Medicine of University of Electronic Science and Technology of China, Chengdu, China

**Keywords:** PI3K, mTOR, dual inhibitor, tumour, PI3K-Akt-mTOR pathway, cancer treament, SAR

## Abstract

The PI3K-Akt-mTOR pathway is a viable target for cancer treatment and can be used to treat various malignant tumours, including follicular lymphoma and breast cancer. Both enzymes, PI3K and mTOR, are critical in this pathway. Hence, in recent years, an array of inhibitors targeting these two targets have been studied, showing dual PI3K/mTOR inhibition compared with single targeting small molecule inhibitors. Inhibitors not only inhibit cell proliferation but also promote cell apoptosis. These inhibitors show high potency and little drug resistance even at low doses, suggesting that PI3K/mTOR inhibitors are promising cancer drugs. Herein, we summarised the recent research of PI3K/mTOR dual inhibitors—for example, structure-activity relationship, pharmacokinetics, and clinical practice, and briefly commented on them.

**Clinical Trial Registration:** https://clinicaltrials.gov.

## Graphical Abstract

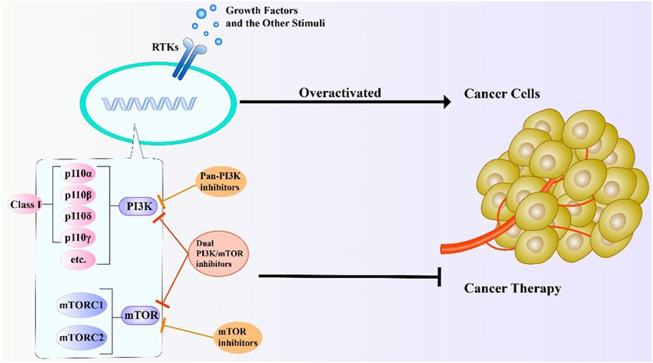

## 1 Introduction

The underlying mechanism of cancer is characterized by complex aberrations that activate key cell signalling pathways. The PI3K-Akt-mTOR signalling pathway is among the most common intracellular signalling pathways that are often abnormally activated in many human cancers and participate in different biological effects, such as cell cycle progression and cell proliferation (Wong et al., 2010; Shimizu et al., 2012). Activation of the PI3K/Akt/mTOR pathway mediated by molecular aberrations plays a crucial role in promoting tumour development and resistance to anticancer therapies. Therefore, this pathway is widely exploited in cancer treatment.

**FIGURE 1:**
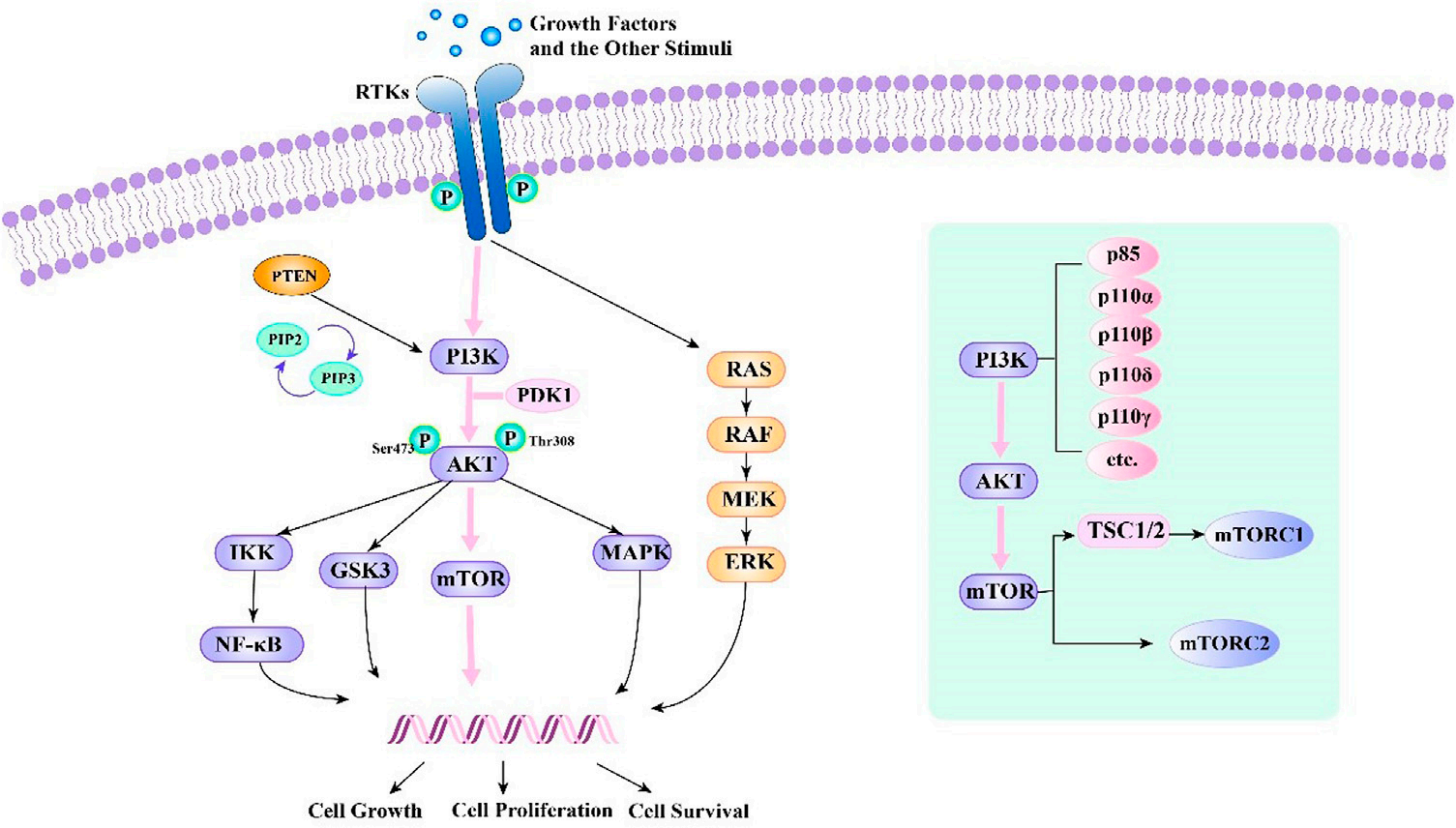
PI3K-Akt-mTOR signalling pathway and relationships of several related signalling pathways.

Growth factors and other stimuli stimulate cells, and they activate receptor tyrosine kinases (RTKs), G protein-coupled receptors, Ras protein, and PI3K kinases (Dienstmann et al., 2014; Thorpe et al., 2015). Then, PI3K kinase produces phosphorylated phosphatidylinositol from phosphatidylinositol. With the participation of phosphatidylinositol-dependent protein kinase-1 (PDK1), PI3K kinase activates it by binding to Akt’s pH domain (Dienstmann et al., 2014). After that, Akt can activate mTOR kinase by phosphorylating mTOR molecules or the TSC1/2 complex, respectively. Afterwards, mTOR kinase activates downstream molecules (Guertin and Sabatini, 2007) (Figure 1).

PI3Ks are enzymes of the PI3K-Akt-mTOR pathway with unique substrate specificity, expression modes, and regulation modes. These enzymes play central roles in regulating cell metabolism, proliferation, and survival (Liu et al., 2009; Thorpe et al., 2015). Activation of PI3Ks initiates signal transduction pathways, which stimulate differentiation, metabolism, migration, cell proliferation, and survival (Hernandez-Aya and Gonzalez-Angulo, 2011). PI3Ks are divided into several classes: class I PI3Ks comprises one regulatory subunit (p85, p101, or p87) and four catalytic subunits: p110α, p110β, p110δ, and p110γ(Katso et al., 2001; Thorpe et al., 2015).

mTOR is a cytoplasmic serine-threonine kinase and a member of the PI3K-related kinase family (PIKKs). mTOR is a key regulator of cell metabolism, growth and survival and can respond to carcinogenic factors (Corradetti and Guan, 2006; Shimobayashi and Hall, 2014). The mTOR protein is at the centre of the PI3K-Akt-mTOR cascade. Two different mTOR complexes have been identified—mTOR complex 1 (mTORC1) and mTOR complex 2 (mTORC2). The former regulates processes such as protein biosynthesis, while the latter fully activates Akt kinase by phosphorylating it at Serine473 (S473). A vital target for cancer therapy, mTOR activation occurs often in human tumours (Loewith et al., 2002; Guertin and Sabatini, 2007; Laplante and Sabatini, 2012; Saxton and Sabatini, 2017; Chen and Zhou, 2020).

**FIGURE 2:**
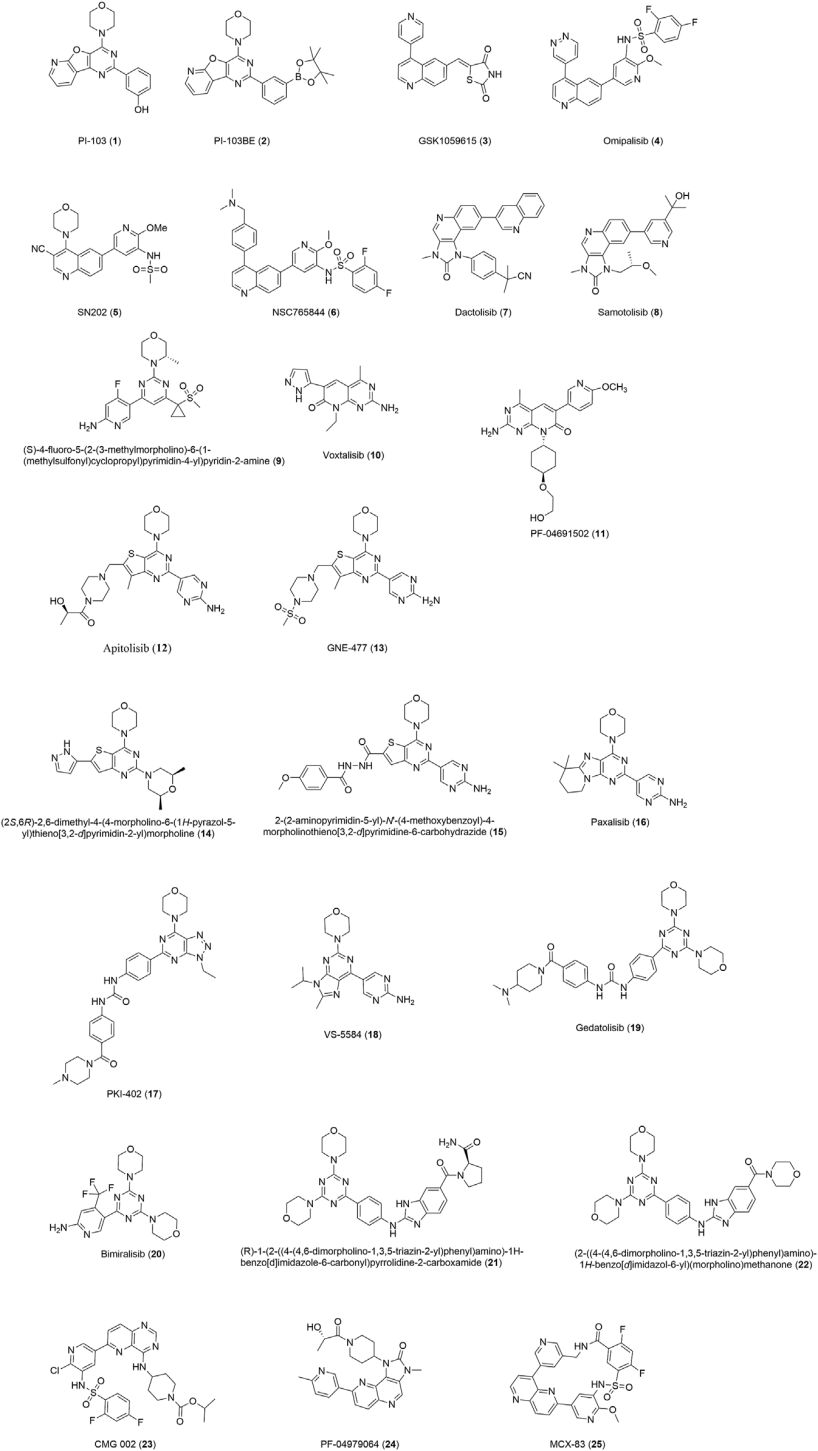
Structures of dual PI3K/mTOR inhibitors mentioned in the article.

Dual PI3K/mTOR inhibitors (Figure 2) show potent activity on all p110 isoforms and mTOR, combining multiple therapeutic effects in a single molecule (Blachly and Baiocchi, 2014). Compared with other types of PI3K pathway inhibitors, PI3K/mTOR dual inhibitors target all catalytic forms of PI3K, as well as of mTORC1 and mTORC2, and can effectively overcome the feedback inhibition observed when mTORC1 inhibitors are used alone. PI3K/mTOR dual inhibitors are far more effective than those targeting only a single protein (Fan et al., 2006; Carnero, 2009). An increasing number of inhibitors of important proteins of the pathway (including PI3K and mTOR) have been targeted, some of which have been approved by the FDA or have entered advanced CTs.

## 2 Pyridofuranopyrimidine

### 2.1 PI-103 (1); PI-103BE (2)

#### 2.1.1 PI-103 is Pyrido [3′, 2′:4,5] Furo [3,2-*d*] Pyrimidine

PI-103, a synthetic small molecule of the pyridofuropyrimidine class, is a potent PI3K/mTOR inhibitor, particularly against class IA enzymes (IC_50_ values for PI3Kα, PI3Kγ, mTOR: 8.4, 86, and 5.7 nM, respectively). However, its metabolism *in vivo* is way too fast, hampering its further clinical development and its ability to enter clinical trials. Yet many studies have used the compound as an exploratory tool to synthesize other PI3K/mTOR inhibitors (Raynaud et al., 2007).

Hayakawa et al. have previously reported 3-(4-morpholinothieno [3,2-d ] pyrimidin-2-yl) phenol, a p110α inhibitor, but its half-life is shorter than 10 min and it cannot be used *in vivo*. Thus, they have identified a similar compound, 4-(pyrido [3′,2′:4,5] thieno[3,2-d] pyrimidin-4-yl) morpholine, which was used as a lead compound and further optimized to obtain PI-103 (Hayakawa et al., 2007).

PI-103 is highly selective for the PI3K superfamily. The IC_50_ values of it, for recombinant isoforms of PI3K, are fairly low (IC_50_ of p110α, p110β, p110δ, and p110γ are 2, 3and 15 nM, respectively). PI-103 was also active against DNA-PK (IC_50_ = 14 nM) with an 83.9% inhibitory effect at 0.5 μmol/L. PI-103 may be developed as a potential therapeutic agent for patients with oral squamous cell carcinoma related to excessive activation of the PI3K/Akt pathway. Additionally, studies have shown that PI-103 exhibits radiosensitisation in PTEN-mutated U251 cells compared with PTEN wild-type T98G cells (Djuzenova et al., 2016). Further, to overcome the shortcomings of the faster metabolism of PI-103 *in vivo*, Luo et al. (2020) designed and synthesized the PI-103 biological isostere PI-103BE. *In vitro*, compared with PI-103, PI-103BE has antiproliferative activity against a group of cancer cell lines, but with lower effectiveness. Though *in vivo*, pharmacokinetic studies in mice have shown that the bioavailability of PI-103BE was significantly improved, and PI-103BE inhibits the growth of xenograft tumours (Luo et al., 2020). These findings might provide new directions for future research.

## 3 Compounds With Quinoline Core

### 3.1 GSK1059615 (GSK615) (3)

**FIGURE 3:**
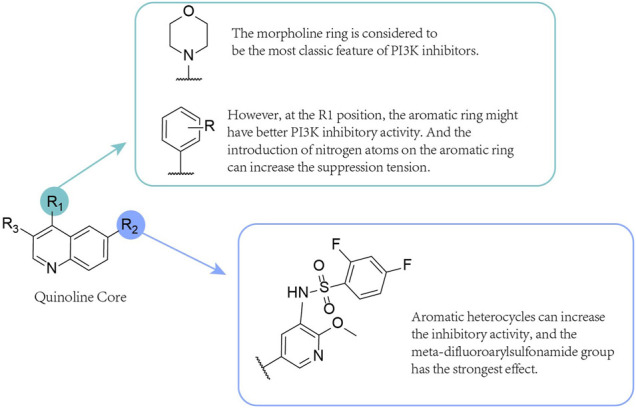
SAR of Quinoline core compounds.

The pyridinylquinoline derivative GSK1059615 (Figure 3) (Leontieva and Blagosklonny, 2016) (also known as GSK615) is a pan-PI3K reversible inhibitor and a pan inhibitor of mTOR (Carnero, 2009; Maira et al., 2010). It has subnanomolar IC_50_ values for PI3Kα and its carcinogenic mutants and PI3Kβ and has a low nanomolar activity for γ, δ and mTOR. The IC_50_ values for PI3Kα, PI3Kβ, PI3Kγ, PI3Kδ and mTOR were 0.4, 0.6, 5, 2 and 12 nM, respectively (Carnero, 2009).

Blockade of the PI3K-Akt-mTOR pathway can effectively inhibit the progression of gastric cancer cells *in vitro* and *in vivo* (Bei et al., 2019, 1059615). Current research findings indicate that GSK1059615 may be a possible anti-gastric cancer drug. In AGS cells and primary human GC cells, GSK1059615 inhibited the growth, survival, proliferation, and cell cycle progression of GC cells and simultaneously induced noticeable apoptosis activation (Bei et al., 2019, 1059615). Notably, no cytotoxicity was observed when GSK1059615 was added to primary human gastric epithelial cells. GSK1059615 blocks the entire PI3K-Akt-mTOR cascade in human GC cells at the molecular level. GSK1059615 was also reported to block mTORC1 (pS6K1) and mTORC2 (p-Akt Ser473) in established and major GC cells. The drug also blocked the activation of PI3K-Akt in GC cells. These findings may explain the excellent anti-GC cell activity of the compound that is more effective than that of AZD2014 and MK2206 (Hirai et al., 2010; Pike et al., 2013). These findings may also explain why GSK1059615 is ineffective in gastric epithelial cells, where the basic activation of the PI3K-Akt-mTOR pathway is sorely low. GSK1059615 treatment was demonstrated to induce miR-9 downregulation and increase the expression of LIM homeobox transcription factor 1α(LMX1A) in established and primary GC cells. Additionally, targeting the miR-9-LMX1A axis may be another critical advantage of GSK1059615 in inhibiting GC cells (Saxton and Sabatini, 2017; Bei et al., 2019, 1059615). The underlying mechanism may require further research.

Furthermore, GSK1059615 was reported to activate PI3K-Akt-mTOR in HNSCC cells but does not cause apoptosis in these cells; instead programmed necrosis occurs (Xie et al., 2017). Interestingly, genetic evidence further confirmed that programmed necrosis mediates GSK1059615-induced cytotoxicity of SCC-9 cells. This activity is relatively rare among PI3K/mTOR dual inhibitors, although various anticancer agents, such as curcumin and cisplatin, can trigger the nonapoptotic death of cancer cells (Chen et al., 2013; Qiu et al., 2014; Xie et al., 2017).

### 3.2 Omipalisib (GSK2126458) (4)

Omipalisib, 2,4-difluoro-N-{2-(methyloxy)-5-[4-(4-pyridazinyl)-6-quinolinyl]-3-pyridinyl}benzenesulfonamide (Figure 3) is a potent inhibitor of PI3Kα (p110α/p85α) (PI3Kα IC_50_ = 0.04 nM) (Knight et al., 2010). It has been identified as the most effective PI3Kα inhibitor reported to date, with an activity 100 times greater than that of dactolisib (IC_50_ = 6 nM) (Maira et al., 2008). The drug shows excellent selectivity for protein kinases, except for the class IV PI3K family, and is also an effective inhibitor of class IV PI3K and DNA-PK (IC_50_ = 0.28 nM). Omipalisib shows high inhibitory activity against PI3Ks and mTOR (mTORC1 app Ki and mTORC2 app K_i_: 0.18 and 0.3 nM, respectively) but has low water solubility and toxicity (Knight et al., 2010).

Knight et al. tried to synthesize a better inhibitor in various aspects using GSK1059615. In the ATP binding pocket, the thiazolidinedione (TZD) ring interacts with the catalytic lysine (Lys833), showing that there is room for larger groups as well. Inhibitors with enhanced potency and selectivity may be developed by filling the empty areas in enzyme pockets. The pyridyl group of 3-methylpyridine and indazole group of 5-methyl-1H-indazole are each likely to interact uniquely with the enzyme. Therefore, the two heterocycles were merged to form an azaindazole (5-methyl-1H-pyrazolo [3,4-b] pyridine, 5-methyl-1H-pyrazolo [3,4-b] pyridine), greatly improving the biochemical and cellular potency. The compound shows very low water solubility and a poor PK profile. Studies have found that arylsulfonamides exhibit oral exposure in rats without loss of inhibitory activity (Knight et al., 2010). Removing the 2-amino group and reversing the sulfonamide connectivity to obtain 2-amino-N-(2,4-difluorophenyl)-5-methylpyridine-3-sulfonamide led to increased biochemical and cellular potency and a noticeable improvement in oral exposure. The reintroduction of small substituents (for example, methoxy, methyl, and halogen) at the 2-position of pyridine leads to a significant increase in enzyme and cell efficiency. Incorporation of pyridazine at the 4-position of quinoline to obtain omipalisib can moderately improve Cytochromes P450 (CYP) inhibitory properties compared with pyridine at the 4-position.

Basu et al. (2018) found that omipalisib treatment can induce autophagy-mediated cell death in neurocutaneous melanoma (NCM) cell death in a dose-dependent manner, an activity that can be inhibited by 5 mM 3-methyladenine (3 MA) (an autophagy inhibitor), supporting autophagy’s role in omipalisib-mediated cell death. Data from Xiao et al. (2020) showed that the PI3K/Akt/mTOR pathway is strongly activated in ovarian tumours in elderly patients. OMIPALISIB is very likely to be the choice for further studies on therapeutic ovarian cancer.

### 3.3 SN202 (5)

SN202 (Figure 3) is a structurally novel compound first discovered to target PI3K/mTOR kinase, a dual inhibitor of PI3K/mTOR with ATP competitive ability (Wang et al., 2018). SN202 significantly inhibits the activities of PI3Kα and PI3Kγ and is expected to be used in further preclinical and clinical development to study its therapeutic value in renal cell carcinoma (RCC) (Husseinzadeh and Garcia, 2011; Pal and Quinn, 2013) (SN202 inhibits PI3Kα, PI3Kγ, and mTOR, and the corresponding IC_50_ values are 3.2, 3.3, and 1.2 nM, respectively). However, it remains unclear how SN202 inhibits PI3Kβ and δ in an isoform-selective manner. It should be noted that the ATP binding sites of most kinases are highly conserved, and therefore, inhibitors developed based on these domains may exhibit weak selectivity and strong cytotoxicity. Additionally, SN202 must be evaluated for different kinase selectivity profiles, and a study of 243 clinical kinase inhibitors found that the number of targets for a particular drug varies greatly (Klaeger et al., 2017). In RCC, PI3K overexpression has been proven to be crucial for tumour progression, and activation of PI3K protein is significantly related to the shortening of the survival time (Elfiky et al., 2011; Cheng et al., 2013). The antitumour efficacy of SN202 was demonstrated in a preclinical kidney cancer xenograft model, showing a dose-dependent tumour growth inhibitory effect but did not cause weight loss, and the dose range relative to mice was wide. After oral administration to rats, good safety and exposure degree were also observed. However, a more comprehensive security assessment of SN202 is still needed. Overall, *in vitro* and *in vivo* experiments have shown that SN202 has a significant antitumour effect on RCC (Elfiky et al., 2011). Additionally, the compound has been found to show safe and positive pharmacokinetic properties in preclinical studies. SN202 is expected to be used in further preclinical and clinical studies to determine its therapeutic value for treating renal cell carcinoma.

### 3.4 NSC765844 (6)

Because p110 and mTOR have similar structural features, PI3K/mTOR dual inhibitors can be designed using their similarity (Frédérick and Denny, 2008; Han and Zhang, 2010; Sabbah et al., 2010).

NSC765844 (Figure 3), a compound synthesized on the arylsulfonamide scaffold as a structurally optimized core structure, is an oral, highly potent and effective dual PI3K/mTOR inhibitor with low toxicity [IC_50_ values of 1.3, 1.8, 1.5, 3.8 and 3.8 nM for PI3Kα, β, γ, δ, and mTOR, respectively (Han et al., 2016)]. Han et al. (2016) found that the arylsulfonamide scaffold is a common privileged structure in PI3K/mTOR inhibitors. For example, omipalisib, voxtalisib, and apitolisib all contain this structure. As shown by crystal structures, several existing inhibitors including p110γ are suitable for the active site of quinolinyl-3-pyridylbenzenesulfonamide. PI3K inhibitors possessing this structure are highly effective against the class I PI3K family as well as mTOR. Therefore, they chose an arylsulfonamide structure as the initial lead scaffold for optimisation. After many syntheses, N-(5-(4-(4-((diethylamino)methyl)phenyl)quinolin-6-yl)-2-methoxypyridin-3-yl)-2,4-difluorobenzenesulfonamide was obtained. It was further optimized to obtain NSC765844, which showed the most effective cytotoxic activity in this series of compounds. NSC765844 has been identified as a potential anticancer drug candidate that warrants further study preclinically and in clinical trials.

## 4 Imidazoloquinoline

### 4.1 Dactolisib (NVP-BEZ235) (7)

#### 4.1.1 Dactolisib (NVP-BEZ235) is an imidazo ([*4*,*5-c*)]quinoline derivative

It is a widely studied PI3K/mTOR inhibitor and an orally available clinical candidate with well-tolerated antitumour activity. It shows a high degree of inhibitory activity against four PI3K paralogues and is also active against the most common type of PI3Kα mutants and mTOR. Because PI3K and mTORC2 are blocked at the same time, a lower IC_50_ value was obtained at the level of Serine473 (Maira et al., 2008). Cell proliferation in cancer cells is stimulated by mTOR signalling in two mechanisms: by activating Akt through mTORC2 and via downregulating mTORC1 at the level of 4E-BP1 (Jacinto et al., 2006). The use of dactolisib alone may cause disease stagnation, but when used in joint with other cancer drugs, it may enhance the effectiveness of the other drugs. The imidazo [4,5-c] quinoline framework can use several binding modes to simulate the interaction between the adenine part of ATP and the H bond of the hinge region. This unique function makes the chemical template a structure-based drug design and an attractive starting point for work (Stauffer et al., 2008).

PDK2 activity occurs primarily because of the mTORC2 complex, and the enzyme does not affect Thr308 phosphorylation (Shiota et al., 2006; Kalaitzidis et al., 2012). However, the lower IC_50_ values obtained for Ser473 levels are unlikely caused by the sole effect on PI3K but the concomitant blockade of PI3K and mTORC2. Dactolisib is more effective than LY294002, which only inhibits PI3K, and can prevent Akt activation, further reflecting the dual inhibition of PI3K and mTORC2. Dactolisib selectively inhibits the PI3K/mTOR pathway.

Dactolisib effectively inhibited the growth of the HCT15 colorectal cancer (CRC) cell line with a nanomolar IC_50_ (Oh et al., 2016). Dactolisib inhibits both the mTORC1 and mTORC2 forms of the mTOR protein. mTORC1 and mTORC2 phosphorylate Akt at Thr308 and Ser473, respectively. However, mTORC1 inhibition is usually related to mTORC2 activation, causing subsequent phosphorylation of Akt at Ser473 to cause significant autophagy in such cells. Additionally, dactolisib induces autophagy, and inhibiting autophagy can lead to salvage of cell viability, indicating that autophagy can destroy cells. The discovery sheds light on the role of autophagy as a mode of cell death in the field of CRC treatment (Maira et al., 2008; Xu et al., 2011; Schult et al., 2012; Kuger et al., 2015; Oh et al., 2016).

Alqurashi and others also proved that dactolisib does not affect the potential of mitochondria to affect cell apoptosis (Alqurashi et al., 2018, 1). Cai et al. observed the downregulation of mutp53 in triple-negative breast cancer (TNBC) cells after dactolisib treatment for the first time (Cai et al., 2020). Dactolisib may be beneficial to cancer patients carrying mutp53 since it has the ability to inhibit proliferation, metastasis, and colonisation of TNBC cells by targeting mutp53. Dactolisib will have an antitumour effect by degrading mutp53, thus it may be a new type of cancer treatment drug. The precise interaction between mutp53 and autophagy must be studied in depth because regulating their interaction will help treat cancer patients in the future.

### 4.2 Samotolisib (LY3023414) (8)

A highly soluble compound over a wide pH range, Samotolisib (Table 1) is an ATP-competitive inhibitor that effectively inhibits PI3K, mTOR, and DNA-PK, as well as inhibiting the viability of cancer cells and causing cell cycle effects. With high oral availability, it inhibits class I PI3K subtypes and mTOR kinase (Smith et al., 2016). Samotolisib effectively and selectively inhibits the three kinases at low nanomolar concentrations, showing high bioavailability. *In vitro*, the inhibitory effect of samotolisib on PI3K/Akt/mTOR signalling leads to G_1_ cell cycle arrest and produces extensive antiproliferative activity in cancer cell screening. Notably, in multiple xenograft models, the equivalent total daily dose of samotolisib once or twice a day inhibited tumour growth to a similar degree, suggesting that intermittent target inhibition could achieve antitumour effects. When combined with standard-of-care drugs, the antitumour activity of samotolisib can be enhanced (Fiebig et al., 2004).

**TABLE 1:** Dual PI3K/mTOR inhibitors in current clinical trials that listed in the article.

Inhibitor	Conditions	Phase	Status	Time period	Intervention model	NCT number	References
Samotolisib	Advanced malignant solid neoplasm	2	Recruiting	24 July 2017 to 30 September 2027	Parallel assignment	NCT03155620	Azaro et al. (2021)
	Ann Arbor stage III non-hodgkin lymphoma						
	Ann Arbor stage IV non-hodgkin lymphoma (and 42 more)						
	Endometrial cancer recurrent endometrial cancer	2	Active, not recruiting	September 2015 to September 2022	Single group assignment (Samotolisib)	NCT02549989	
	Triple negative breast cancer	2	Recruiting	5 September 2019 to August 2022	Single group assignmen (1. samotolisib 2. Prexasertib)	NCT04032080	
	Breast neoplasms	1	Active, not recruiting	10 March 2014 to 28 October 2022	Parallel assignment	NCT02057133	
	Solid tumor breast cancer colon cancer cholangiocarcinoma soft tissue sarcoma	1b	Completed	4 November 2016 to 13 February 2020	Parallel assignment	NCT02784795	
Apitolisib	Prostate cancer	1/2	Active, not recruiting	11 January 2012 to 30 April 2021*	Parallel assignment	NCT01485861	
Paxalisib	Primary central nervous system lymphoma non-hodgkin lymphoma of extranodal site	2	Recruiting	1 June 2021 to 1 May 2024	Single group assignment (Paxalisib)	NCT04906096	
	Glioblastoma, adult	2	Active, not recruiting	15 May 2018 to 30 December 2020*	Single group assignment (Paxalisib)	NCT03522298	
	CDK gene mutation metastatic malignant neoplasm in the brain metastatic malignant solid neoplasm (and 3 more)	2	Recruiting	15 August 2019 to June 2025	Parallel assignment	NCT03994796	
	Glioblastoma	2/3	Recruiting	30 July 2019 to June 2024	Sequential assignment	NCT03970447	
	Brain metastases leptomeningeal metastasis	1	Recruiting	6 December 2019 to December 2022	Single Group assignment	NCT04192981	
	Brain and central nervous system tumours	1	Active, not recruiting	19 November 2018 to June 2024	Parallel assignment	NCT03696355	
	Breast cancer	2	Recruiting	11 February 2019 to 30 November 2025	Parallel assignment (1. Trastuzumab, 2. Paxalisib)	NCT03765983	
Gedatolisib	HER2-positive breast cancer metastatic breast cancer	2	Recruiting	3 December 2019 to December 2021	Single group assignment (1. Trastuzumab biosimilars (Herzuma), 2. Gedatolisib)	NCT03698383	
	TNBC - Triple-negative breast cancer	1/2	Recruiting	17 April 2019 to May 2022	Single group assignment (1. Gedatolisib 2.Talazoparib)	NCT03911973	
	Lung cancer squamous Cell Solid tumours head and neck cancer pancreatic cancer	1	Recruiting	28 February 2017 to January 2023	Single group assignment (1. Palbociclib 2. Gedatolisib)	NCT03065062	
	Breast cancer	1	Active, not recruiting	January 2016 to September 2020*	Single group assignment	NCT02626507	
	Triple negative breast cancer metastatic breast cancer	1	Completed	19 January 2018 to 27 May 2020	Sequential assignment (1.Gedatolisib 2.PTK7-ADC)	NCT03243331	
	Breast cancer	1b	Completed	14 June 2016 to 11 June 2022	Parallel assignment	NCT02684032	Forero et al. (2018)
	Neoplasm	1b	Completed	September 10, to 2013 8 January 2020	Single group assignment	NCT01920061	
Bimiralisib	HNSCC	2	Terminated	25 January 2019 to 5 August 2020	Single group assignment	NCT03740100	Johnson et al. (2020)
	Lymphoma, Malignant	2	Completed	May 2015 to 11 September 2018	Single group assignment	NCT02249429	
	Primary central nervous system lymphoma	2	Completed	12 November 2015 to 12 January 2018	Single group assignment	NCT02669511	

The data were collected from https://clinicaltrials.gov (Last accessed 23 Mar 2022).

Biliary tract carcinoma (BTC) is a tumour that has aggressive characteristics. In Sakamoto’s research, samotolisib inhibited the growth of the BTC cell lines NCC-BD2, NCC-BD3 and OZ, despite the resistance to gemcitabine. The inhibition of proliferation by samotolisib was nearly complete in the NCC-BD2 and NCC-BD3 cell lines (Smith et al., 2016; Sakamoto et al., 2018). This study shows that using a preclinical model of the BTC cell line will provide a novel method for screening a variety of drugs *in vitro* and suggests that PI3K/mTOR inhibitors may be new drugs for BTC drug development.

Human cancer cells have been investigated for potential effects of overexpressing ATP-binding cassette (ABC) drug transporters on samotolisib efficacy (Shukla et al., 2011; Wu CP. et al., 2020). Wu et al.’s work has somewhat demonstrated that, as a result of inhibiting ABCB1 and ABCG2, samotolisib can be restored the efficacy in ABCB1- and ABCG2-overexpressing multidrug-resistant cancer cells. They also concluded that samotolisib is a substrate of ABCB1 and ABCG2 because it stimulates the ATPase activity of the two. Yet, considering the influence of ABCB1 and ABCG2 on the absorption and distribution of therapeutic drugs, samotolisib transport mediated by ABCB1 and ABCG2 may pose a major therapeutic challenge to clinicians in the future, so does the bioavailability. Hence, down to problems such as toxicity and weak metabolic stability of inhibitors, there is no FDA-greenlit treatment currently. Further research is in need to explore viable methods for drug combinations that may overcome ABCB1- and ABCG2-mediated resistance to samotolisib.

## 5 Pyrimidine

### 5.1 2-Morpholino-Pyrimidine Derivatives Containing Multiple Sulfonyl Side Chains at the C4 Position (9)

The IUPAC name of Compound **9** is (S)-4-fluoro-5{[2-(3-methylmorpholino)-6-(1-(methylsulfonyl)cyclopropyl]pyrimidin-4-yl}pyridin-2-amine (Shen et al., 2018).

Buparlisib (also known as NVP-BKM120 or BKM120) is a brain-penetrable, orally available pan Class Ⅰ PI3K inhibitor (Burger et al., 2011). By substituting a sulfonyl moiety selected from selective mTOR inhibitors for the C4 morpholine moiety on buparlisib, a novel PI3K inhibitor, Compound 9, based on morpholinopyrimidine was discovered. The binding mode of Compound 9 and PI3Kα is very similar to that of buparlisib and has similar potency to PI3Kα.

The compound is a potent dual PI3K/mTOR inhibitor. Fluorine, although it does not have a significant antiproliferative effect, is the reason for the increased mTOR activity of the C6 aminopyridyl moiety. The researchers compared the C6 aminopyridyl moiety with trifluoromethyl and fluorine (that is, the compound) and found that when fluorine was used, the potency against mTOR was increased 11 times, and the potency against the PI3Kd subtype was increased 4 times. In HT-29 cells, the drug slightly inhibited the ATR cell activity of Chk1 Ser345 phosphorylation at a concentration of 10 μM. The compound is superb in oral bioavailability. Compared with buparlisib, in the HT-29 colorectal cancer xenograft mouse model, Compound 9 also exhibits satisfactory ADMET properties and outstanding tumour growth inhibitory effects *in vivo*.

## 6 Pyridopyrimidinone Derivative

### 6.1 Voxtalisib (XL765, SAR245409) (10)

Voxtalisib is a pyridopyrimidinone derivative, a highly selective pan inhibitor of class I PI3Ks with inhibiting efficacy mTOR.

As a compound identified after optimizing the pyridopyrimidinone scaffold for inhibiting PI3K/mTOR pathway and drug-like attributes *in vivo*, voxtalisib is the first orally available PI3K/mTOR dual inhibitor that is effective and selective and an ATP competitive reversible inhibitor (Yu et al., 2014). It has remarkable inhibitory activity against PI3K subtypes p110α, p110β, p110δ and p120γ of class I (IC_50_ values for PI3Kα, PI3Kβ, PI3Kδ, PI3Kγ, VPS34, mTORC1, mTORC2, DNA-PK: 39, 110, 43, 9, 9,060, 160, 910, and 150 nM, respectively) (Yu et al., 2014). Among the voxtalisib-interacting residues of PI3Kγ, Lys-890 and Met-953 are precisely positioned as the key residues required for binding (Rehan, 2019). Regarding mTOR, Trp-2239 is positioned as the key interaction residue. Another residue, Asp-2251, uses the N-amino atom of the guanidine group to form a hydrogen bond with one of the N-atoms of the quinoxaline moiety of the voxtalisib interaction. Six new compounds were obtained by modifying the voxtalisib scaffold. These compounds have passed most of the drug similarity and pharmacokinetic property tests, indicating that these six new compounds can perform as safe candidates for human use. Additionally, enrichment analysis indicated the selective binding and quality binding with the targeted PI3Kγ and mTOR. The analysis showed that these six new compounds are better than the starting compound voxtalisib as dual PI3K/mTOR inhibitors. Therefore, the current docking analysis of voxtalisib with PI3Kγ and mTOR will provide an excellent model to study the molecular interaction of drug-protein complexes (where the drug targets multiple proteins) and will also help the future design of novel and effective medicine (Rehan, 2019).

Voxtalisib has a wide range of anticancer efficacy and controllable safety in patients with advanced solid tumours (Papadopoulos et al., 2014). The study by Gravina et al. showed that voxtalisib inhibits the phosphorylation of p70S6 kinase. Compared with inhibiting PI3K or mTOR alone, voxtalisib is far more effective in reducing the concentration-dependent reduction of viable/proliferating tumour cells (Gravina et al., 2016). Zhang et al. showed that voxtalisib dose-dependently inhibited the proliferation of four leukaemia cell lines with IC_50_ values less than 5 μM. By contrast, voxtalisib has a weak inhibitory effect on normal human peripheral blood mononuclear cells, with an IC_50_ of 43.43 μM, reflecting its selectivity. Voxtalisib induced G1 cell cycle arrest and did not induce significant apoptosis in these four cell lines. Voxtalisib can reduce the expression of MDR1 and MRP1 in HL60/ADR and K562/A02 cells, indicating that voxtalisib may reduce MDR1 and MRP1 expression to reverse the MDR of these two cells. These results indicate that voxtalisib may treat leukaemia (Zhang et al., 2018). Additionally, research data have indicated that voxtalisib-targeted inhibition of PI3K/mTOR is a promising treatment strategy to reduce the tumour burden of patients with glioblastoma (GBM) (Zhao et al., 2019).

### 6.2 PF-04691502 (11)

PF-04691502 is a compound derived from the 4-methylpyridopyrimidinone series {2-amino-8-[trans-4-(2-hydroxyethoxy)cyclohexyl]-6-(6-methoxypyridin-3-yl)-4-methylpyrido- [2,3-d]pyrimidin-7(8H)-one}.

PF-04691502 is a PI3K inhibitor with PI3K/mTOR inhibitory effects and shows various antitumour effects (Yuan et al., 2011). As measured by the PI3K-independent nutrient stimulation assay, with an IC_50_ of 32 nM, PF-04691502 inhibits mTORC1 activity in cells and inhibits the activation of PI3K and/or mTOR downstream effectors, such as FKHRL1, PRAS40, 4EBP1, and so forth. PF-04691502 inhibited human and mouse PI3Kα with Ki values of 1.8 and 1.2 nM, respectively, PI3K isoforms β, δ, and γ with Ki values of 2.1, 1.6, and 1.9 nM, respectively, and human mTOR with Ki values of 16 nM. Langdon et al. used two PI3K/mTOR inhibitors, gedatolisib and PF-04691502, to study six human patient-derived ovarian cancer xenograft models and found that both compounds exhibit antitumour activity on all the experimental substances, and the initial tumour volume inhibitory effect seems to be the strongest. The study has shown, however, that there is drug dependence—continuous administration of these inhibitors is active, yet stopping treatment will cause tumours to grow again (Langdon et al., 2019). These findings, when used as maintenance therapy after chemotherapy, may be valuable for prolonging the progression of the disease.

## 7 Thienopyrimidine Core

### 7.1 Apitolisib (GDC-0980) (12)

**FIGURE 4:**
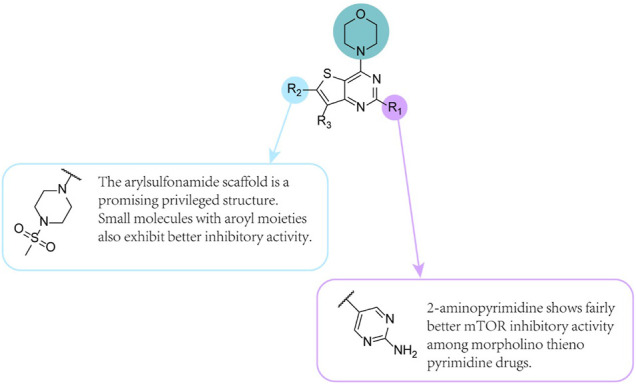
SAR of thienopyrimidine core compounds.

Apitolisib (Table 1, Figure 4) is a compound that uses the lead compound pictillisib as a structural template and replaces indazole with 2-aminopyrimidine (Folkes et al., 2008). It is an effective and selective class I PI3K/mTOR inhibitor (Sutherlin et al., 2011). The structure of the quinoline ring is common in PI3K inhibitors. Among the compounds with different substituents introduced at the C-4 position of the quinoline ring, pyrrolidine derivatives showed significant loss in inhibiting PI3Kα activity; morpholine derivatives and p-tolyl substituted derivatives retained high enzyme inhibition. It is active and shows approximately 2 times the *in vitro* cytotoxic activity in the three tumour cell lines.

When the 2-methoxy group is substituted by a chlorine atom, the inhibitory effect of the compound on PI3Kα is equivalent or slightly reduced. Additionally, when the 2,4-difluoro analogue of these compounds is substituted with 4-fluoro, the compound exhibits an equivalent PI3Kα inhibitory effect.

Substituting 2-aminopyrimidine with indazole plays a key role in enhancing the effectiveness of mTOR by an average of 10-fold in many different morpholinothienopyrimidines. Compared with human liver microsomes, this compound has a lower predicted clearance rate in humans, a lower *in vivo* clearance rate in rats, and excellent oral bioavailability. Apitolisib is a small molecule inhibitor of the PI3K/mTOR pathway with effective antiproliferative activity and good solubility. The results of a study provide a basis for the future use of the drug to treat paediatric leukaemia (Al-Ghabkari et al., 2019). In that study, five different leukaemia cell lines and normal cells were used for comparison. After applying GDC-0980, FLT3 phosphorylation in Molm-13 and MV4-11 cells was reduced. And it also showed that using MEK inhibitors and FLT3 inhibitors synergistically can treat childhood leukaemia.

### 7.2 GNE-477 (13)

Heffron et al. identified a novel and potent dual PI3K/mTOR inhibitor, GNE-477 (Figure 4) (Heffron et al., 2010). Similar to previous studies, the drug is synthesized from pictilisib as the starting point (Folkes et al., 2008). When the indazole part of pictilisib was replaced by 2-aminopyrimidine, the demethylated analogue 5-(6-((4-(methylsulfonyl)piperazin-1-yl)methyl)-4-morpholinothieno [3,2 -d]pyrimidin-2-yl) pyrimidin-2-amine maintained its inhibitory effect on PI3K-a and effectively inhibited mTOR kinase activity. This surrogate group has been proven to be effective in identifying many potential PI3K/mTOR dual inhibitors. To improve the clearance rate *in vivo*, this compound was further optimized, and GNE-477 was obtained. Compared with the former, GNE-477 showed superior pharmacokinetic properties and an attractively low clearance rate *in vivo*. Activation of the PI3K-Akt-mTOR pathway is critical for the cellular progression of renal cell carcinoma, but many tested PI3K-mTOR kinase inhibitors failed to significantly improve the clinical symptoms of RCC patients (Husseinzadeh and Garcia, 2011; Pal and Quinn, 2013). Notably, both mTORC1 and mTORC2 are critical to the development of renal cell carcinoma, and mTOR drug inhibitors have shown therapeutic value for RCC (Ye et al., 2020). However, mTORC1 inhibitors may still have some limitations and disadvantages, including incomplete mTOR inhibition and feedback activation of other carcinogenic signals. Ye et al. found that GNE-477 prevented the phosphorylation of p70S6K1-S6 and Akt (S473 and Thr308) in RCC1 cells. Therefore, it inactivates both the mTORC1 and mTORC2 cascades. *In vivo*, GNE-477 was more effective than AZD2014 in inhibiting the growth of RCC1 xenografts (Pike et al., 2013). These results prove that the compound may have significant therapeutic value to treat RCC. Further research, however, is in need to determine the safety and therapeutic effect of this drug. Additionally, the findings showed that GNE-477 does not cause significant toxicity to nude mice (Ye et al., 2020).

### 7.3 (2S,6R)-2,6-Dimethyl-4-{4-Morpholino-6-(1H-Pyrazol-5-yl)Thieno[3,2-d]Pyrimidin-2-yl} Morpholine (14)

(2S, 6R)-2,6-Dimethyl-4-(4-morpholino-6-(1H-pyrazol-5-yl)thieno [3,2-d]pyrimidin-2-yl)morpholine (Figure 4), Compound **14**, is also pictilisib-inspired, a possible candidate dual PI3K/mTOR inhibitor, and has good inhibitory activity (IC_50_ values of PI3Kα and mTOR: 15 and 16 nM, respectively) (Folkes et al., 2008; Zhan et al., 2017).

The thieno [3,2-d]pyrimidine analogues deserve further optimisation, although the mTOR inhibitory activity is unsatisfactory. However, the C6 region has the greatest degree of flexibility in the structure-activity relationship and continues to modify the C-6 position of the thieno [3,2-d]pyrimidine core (Murray et al., 2012).

A characteristic of many PI3K inhibitors is their morpholine rings, and the oxygen atom on the morpholine ring may form a key bond with amino acid residues (Andrs et al., 2015). Several PI3K inhibitors, such as gedatolisib (Structure **19**), are designed to improve solubility or potency by incorporating a second morpholine group into the triazine or pyrimidine core.

Compounds with cis 2,6-dimethyl substituents on the morpholine ring have increased mTOR inhibitory efficacy and maintain PI3Kα inhibitory activity. Notably, compared with ortho- and para-substituted pyridine derivatives, meta-substituted pyridines show promising inhibitory activity against PI3Kα and mTOR.

Compound **14** can mechanically regulate the cellular PI3K/Akt/mTOR pathway by inhibiting the phosphorylation of Akt and S6 in human cancer cell lines. Additionally, Compound **14** has shown measurable efficacy in SKOV-3 and U87MG tumour xenograft models without causing measurable weight loss and toxicity (Zhao et al., 2012).

### 7.4 2-(2-Aminopyrimidin-5-yl)-N'-(4-Methoxybenzoyl)-4-Morpholinothieno (3,2-d)Pyrimidine-6-Carbohydrazide

Han et al. designed four series of novel thieno [3,2- d]-pyrimidine derivatives with aroyl hydrazone or aryl hydrazide moieties (Figure 4) as dual PI3K/mTOR inhibitors for the first time (Han et al., 2020).

Small molecules with aroyl moieties display excellent anticancer activity because of the hydrogen bond donors and acceptors they contain and their flexible structure. Hydrazone and hydrazide fragments are crucial pharmacophores in the field of drug design, thanks to the truth that these groups can easily bind to a wide range of receptors and enzymes through hydrogen bond interactions. The team designed and synthesized two new 2,4-dimorpholinylthieno[3,2-d]pyrimidine derivatives with aroyl and arylhydrazide groups. The findings of these compounds indicate that N′-benzoyl-2,4-dimorpholinothieno[3,2-d]pyrimidine-6-carbohydrazide derivatives are favourable to the inhibitory activity *in vitro* (PI3Kα IC_50_ = 0.46 nM; PI3Kβ IC_50_ = 55 nM; PI3Kγ IC_50_ = 13 nM; PI3Kδ IC_50_ = 32 nM; mTOR IC_50_ = 12 nM). After multiple reactions of 3-aminothiophene-2-carboxylate, 2-chloro-4-morpholino [3,2-d]pyrimidine-6-carboxylic acid is obtained. Compound **15** showed the most effective antiproliferative activity against PI3K and mTOR and showed good inhibitory effects on HCT-116, PC-3, A549 and MDA-MB-231 cell lines. In a word, these reports indicate that the compound can be used as a targeted compound for dual PI3K/mTOR inhibitors. Further study on the compound is warranted.

## 8 Cyclized Thiophenopyrimidine Derivative

### 8.1 Paxalisib (GDC-0084) (16)

Paxalisib (Table 1) is a dual PI3K/mTOR inhibitor, one that can cross the blood-brain barrier (Salphati et al., 2012; Heffron et al., 2016).

Inhibition of PI3K is an effective means to treat GBM. However, to obtain this effect, the drug must cross the blood-brain barrier. Knowing that ideal metabolic stability can be achieved on the purine scaffold, the researchers first studied the Compound 5-(9-methyl-6-morpholino-9H-purin-2-yl) pyrimidin-2-amine, which has a low efflux ratio and excellent stability of human microsomes. Disappointingly, a PC3 cell proliferation test showed deficiency in potency. The researchers further optimized and obtained paxalisib, one that resulted in a high level of metabolic stability, outstanding cell potency, and no efflux. Furthermore, Compound **16** maintains the inhibition of each type I PI3K isoform but has a stronger inhibitory effect on mTOR. Also, Compound **16** was tested in different GBM cell lines, and its antiproliferative EC_50_ values varied from 0.3 to 1.1 μM.

Paxalisib is highly selective for a panel of 229 kinases and has a low clearance rate. Unlike other drugs, it can pass through the blood-brain barrier and shows promising activity in preclinical models of glioblastoma. The drug can inhibit its target after penetrating the blood-brain barrier (Salphati et al., 2012). After oral administration at a dose of 25 mg/kg 16 times, p-Akt in the brain tissue of normal mice was remarkably inhibited 1 and 6 h after administration. In the brain, activation of the PI3K/Akt/mTOR pathway, say, HER3 activation, is believed to mediate resistance to HER2-targeted drugs. Studies have shown that paxalisib has a genotype-selective effect. Compared with wild-type (WT) PIK3CA, paxalisib produces a significant response in the treated PIK3CA-mutant (MT) breast cancer cell line (Ippen et al., 2019). It has been shown that combined inhibition of the PI3K and mTOR pathways can overcome these resistance mechanisms, making them potentially useful in treating affected patients (Ni et al., 2016). Further verification is warranted in clinical trials to determine whether paxalisib can provide therapeutic benefits for patients.

## 9 Triazolopyrimidines

### 9.1 PKI-402 (17)

PKI-402 is a highly potent PI3K/mTOR dual-target inhibitor based on a triazolopyrimidine scaffold (IC_50_ values of PI3Kα, PI3Kγ, and mTOR: 1.4, 9.2, and 1.70 nM, respectively) (Yang et al., 2006; Dehnhardt et al., 2010). Triazolopyrimidine, which shows better potency than imidazolopyrimidine, has been determined using various N-3-substituents; ethyl was found to be the best choice. Various aryl and heteroaryl ureido appendages were screened, and 4-benzamide analogues were found. This group of compounds showed particularly high efficacy on the activity of PI3Kα and mTOR enzymes and had excellent efficacy in tumour cell proliferation tests. Additionally, the IC50 value for MDA-361 cells was much lower than 10 nM, while that for PC3 cells was 21 nM. This compound also exhibits great pharmaceutical properties, say, stability in nude mice and human microsomes and water solubility at pH 3.

PKI-402 was confirmed to inhibit the growth of breast cancer cells (Dehnhardt et al., 2010; Mallon et al., 2010). Research by (Yuan et al., 2019) showed that PKI-402 inhibits the bone resorption of osteoclasts and the formation of filamentous actin (F-actin) loops. The results demonstrated that PKI-402 inhibits the proliferation, invasion and migration of breast cancer cells *in vitro*. Using this inhibitor resulted in a significant reduction in the tumour volume in the bone and reversal of bone destruction. These results strongly indicate that PKI-402 is a possible drug to treat osteolysis caused by breast cancer. (Hu et al., 2020) reported that the PI3K/mTOR dual inhibitor PKI-402 not only inhibits the proliferation of the cisplatin-sensitive ovarian cancer epithelial cell line A2780 but also inhibits the proliferation of the cisplatin-sensitive ovarian cancer epithelial cell line A2780. The cancer epithelial cell line SKOV3 has a significant inhibitory effect. PKI-402 damages mitochondrial function, degrades Mcl-1 protein through autophagy, and induces apoptosis through the mitochondrial pathway. Additionally, this study suggested that the autophagy receptor protein p62 triggers the degradation of Mcl-1 through its UBA domain autophagy.

## 10 Purines

### 10.1 VS-5584 (Formerly Named SB2343) (18)

VS-5584 is a novel low-molecular-weight compound PI3K/mTOR dual inhibitor with almost the same activity for all class I PI3K subtypes and mTOR. mTOR (IC_50_ = 37 nM), PI3Kα (IC_50_ = 16 nM), PI3Kβ (IC_50_ = 68 nM), PI3Kγ (IC_50_ = 25 nM), and PI3Kδ (IC_50_ = 42 nM), without relevant activity on 400 lipid and protein kinases (Hart et al., 2013; Poulsen et al., 2014). This compound is a purine. The small substituent at position eight increased the activity of mTOR and PI3Kα through hydrophobic contacts with residues Ile2163, Ser2165 and Pro2169 in mTOR and Met772 and Ser774 and Pro778 in PI3Kα. Notably, VS-5584 is effective in many rapamycin-resistant cell lines. A single oral dose of VS-5584 is quickly absorbed, with a t_max_ of 0.9 h and an elimination half-life of 10 h. Tumour-bearing PC3 mice were treated with a single dose of VS-5584, and plasma was collected 6 h later and analysed for the tumour VS-5584 concentration and its effect on target efficacy biomarkers. The plasma level of VS-5584 increased in a dose-dependent manner. No significant difference was found between plasma pharmacokinetics and tumour pharmacokinetics. After a single oral administration of VS-5584 in tumour-bearing mice, impressive oral bioavailability in tumour tissues was observed, dose-linear pharmacokinetics, and a deep and lasting effect in tumour tissues. This pharmacokinetic characteristic is critical because it can effectively block mTORC1 and two and PI3K signal transduction in tumour tissues (VS-5584 with mTOR and PI3Kα activities of 37 and 16 nM, respectively).

In the case of relapsed and refractory multiple myeloma (MM), the prognosis is poor. (Mustafa et al., 2017) showed that VS-5584 may provide a longer-lasting chemotherapy response and may become a possible candidate for the combination therapy of relapsed and refractory patients. The reason is that *in vivo*, VS-5584 can significantly reduce the tumour burden of xenografts in MM mice. Additionally, VS-5584 induces the expression of RARRES3 (a class II tumour suppressor gene), a gene that may be useful for the evaluation of the effects of VS-5584 in the clinic. Furthermore, Xu et al. showed that VS-5584 effectively inhibits the proliferation and survival of RCC cells. Targeting inhibition of BRD4 (protein 4 containing bromodomain) can further enhance its antitumour activity (Xu et al., 2020, 5584). As evidenced by both pharmacological and genetic data presented, BRD4 is a VS-5584 resistance factor in renal cell carcinoma (RCC) cells. It might be a crucial strategy to inhibit BRD4 for sensitizing RCC cells to VS-5584.

## 11 Triazine Derivatives

### 11.1 Gedatolisib (PKI-587; PF-05212384) (19)

**FIGURE 5:**
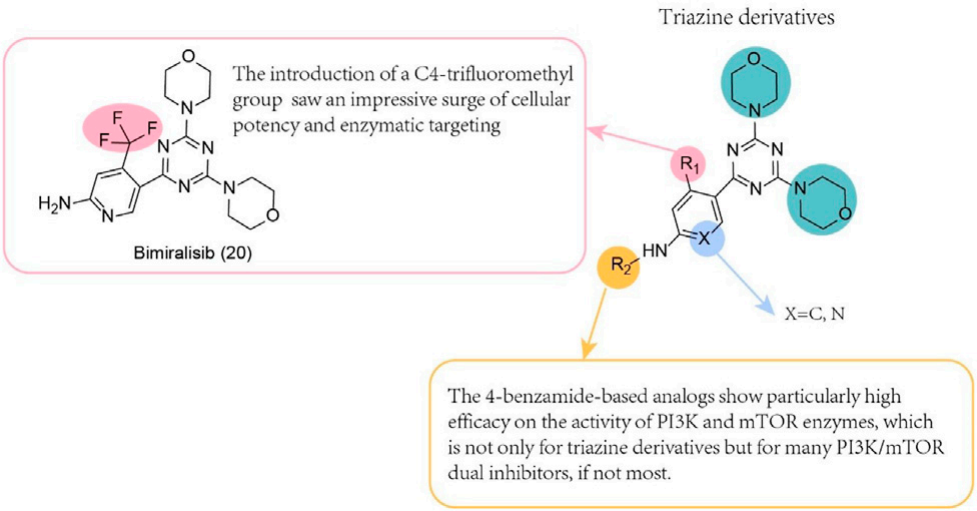
SAR of triazine derivatives.

Gedatolisib (Table 1, Figure 5) is a pan-PI3K/mTOR inhibitor with equivalent potency and high selectivity (Venkatesan et al., 2010).

Several morpholines with fused pyrimidines have been previously reported, say, triazolopyrimidines and imidazolopyrimidines, as PI3K/mTOR dual inhibitor scaffolds. Morpholine oxygen and Val851 of the PI3Kα catalytic domain formed a key hinge region hydrogen bonding, but also through the oxidation of R on morpholine epoxy metabolism, resulting in loss of effectiveness. Many PI3K enzyme inhibitors containing the above scaffolds showed poor antitumour efficacy *in vivo* tests. Among them, Compound 1-(4-chlorophenyl)-3-[4-(4,6-dimorpholin-4-yl 1,3,5-triazin-2-yl)phenyl]urea showed positive efficacy in the MDA-361 xenograft model. Further study of it, however, was stopped because of its poor solubility. Considering these findings, we focused on other stents with similar triazolopyrimidine cores in PKI-402 but whose clogP value is lower than that of the triazolopyrimidine core. Among the various heterocycles synthesized, 1,3,5-triazine scaffolds are considerable. When the phenyl group was substituted by an alkyl group such as a methyl, ethyl or saturated heterocyclic group, the effectiveness of PI3Kα and PI3Kγ was decreased. The activity of mTOR, however, was unaffected. Most compounds with a basic amine moiety with an amide bond exhibit excellent enzyme and cell efficacies.

*In vivo*, gedatolisib has shown antitumour activity (intravenous route) in many cancer xenograft models, including non-small cell lung cancer (Mallon et al., 2011). It has a high proliferation index and a strong tendency for early metastasis. Comprehensive genome analysis showed that the PI3K/Akt/mTOR pathway is a viable therapeutic target in small cell lung cancer (SCLC), a highly aggressive neuroendocrine tumour. Metabolic disorders are a hallmark of cancer. In drug-susceptible but not drug-resistant pulmonary adenocarcinoma cells, treatment with kinase inhibitors may affect the levels of metabolites (Makinoshima et al., 2014). Makinoshima et al. showed that the level of purine-linked metabolites may be a possible biomarker for the response to PI3K/Akt/mTOR-targeted inhibitors (Makinoshima et al., 2018). High levels of purine-related metabolites make small cell lung carcinoma cells resistant to PI3K pathway inhibitors. The team studied PIP, PIP2, and PIP3 through mass spectrometry analysis and found that gedatolisib effectively inhibited the growth of SCLC tumours in tumours. Additionally, the level of metabolic biomarkers can predict the response to the inhibitor.

### 11.2 Bimiralisib (PQR309) (20)

The dimorpholinotriazinyl compound bimiralisib (Table 1, Figure 5) is an effective 4,6-dimorpholino-1,3,5-triazine-based panclass I PI3K inhibitor and an orally bioavailable selective PI3K/mTOR dual inhibitor (Yang et al., 2006; Beaufils et al., 2017). The drug is inspired by 2-(2-difluoromethylbenzimidazol-1-yl)-4,6-dimorpholino-1,3,5-triazine (ZSTK474) (Kong and Yamori, 2007), the final product obtained by the synthesis product. Hepatotoxicity was not found after the oral administration of bimiralisib to female rats for 24 h, but antiproliferative effects *in vitro* and antitumour activity *in vivo* were observed. Bimiralisib also promotes obvious G1 blockade and cell apoptosis in a dose-dependent manner, and cell migration and invasion capabilities are also inhibited. Wound healing, cell migration and cell invasion tests confirmed that bimiralisib reduced the migration and invasion of human glioma cell lines. Yang et al. demonstrated that bimiralisib inhibits the proliferation of GBM U87 and U251 cells and induces their apoptosis, but the drug has limitations. For example, animal experiments are lacking to evaluate whether bimiralisib can be taken orally and crosses the blood-brain barrier in a GBM model, which should be defined and acknowledged (Yang et al., 2020).

Bimiralisib is an orally available, potent selective PI3K/mTOR dual inhibitor that targets mTOR kinase in a balanced manner at higher concentrations (Beaufils et al., 2017). Similar to paxalisib (**16**), the drug can cross the blood-brain barrier. Therefore, bimiralisib has broad prospects as a single drug and a combination drug.

### 11.3 Novel Substituted Triazine

The morpholine part of an orally available pan-class I PI3-kinase inhibitor, NVP-BKM120 (Figure 5), is substituted by various aliphatic or long-chain substituted aromatic amines. These modifications improve the selectivity of PI3K isoenzymes (Burger et al., 2011; Bohnacker et al., 2017). The introduction of a benzimidazole motif in gedatolisib might lead to improved metabolic stability while maintaining biological activity. Therefore, based on the structures of the known anticancer drugs gedatolisib and alpelisib, researchers designed and synthesized a series of newly substituted triazines with benzimidazole scaffolds. The researchers found that the selectivity of PI3K isoenzymes was improved after the morpholine part was replaced by various aliphatic or long-chain substituted aromatic amines. Therefore, a novel substituted triazine derivative of the PI3K/mTOR dual inhibitor benzimidazole scaffold was designed and synthesized.

The hydrogen bonds formed in the hinge domain are essential for inhibiting the activity of PI3Kα and mTOR. The obtained compound was evaluated as a possible dual PI3K/mTOR inhibitor and showed nanomolar kinase inhibitory activity. The selective determination of isozymes showed that they are expected to be dual inhibitors of PI3Kδ/mTOR (R)-1-(2-((4-(4,6-Dimorpholino-1,3,5-triazin-2-yl)phenyl)amino)-1H-benzo [d]imida zole-6- carbonyl)pyrrolidine-2-carboxamide (Compound **21**) and (2-((4-(4,6-Dimorpholino-1,3,5-triazin-2-yl)phenyl)amino)-1H-benzo [d]imidazol- 6-yl) (morpholino)methanone (Compound **22**) (Wu TT. et al., 2020). Western blot analysis revealed that both compounds almost completely blocked the phosphorylation of Akt (p-Akt) at residue serine 473 (S473) and that of p70S6K (p-p70S6K) at residue threonine 389 (T389). Therefore, these two compounds were identified as potential dual PI3K/mTOR inhibitors. PI3Kα and mTOR also form hydrogen bonds with the benzimidazole motif in the inhibitor. Studies on the antiproliferative activity of different human cancer cell lines show, compared to the positive control (gedatolisib), that the synthetic compound has higher activity on HCT116 cells. An additional and more important finding was that the prepared analogues maintained their metabolic stability in man-made gastric juice better than gedatolisib.

## 12 Pyridopyrimidine

### 12.1 CMG002 (23)

Choi et al. developed CMG002, a novel PI3K/mTOR dual inhibitor, and demonstrated that CMG002 is the first-ever compound that inhibits the growth of chemoresistant cancer cells in both vivo and vitro. It also inhibits cell proliferation in chemoproliferative ovarian cancer cells, induces G1 cell cycle arrest and apoptosis, and resensitizes chemoresistant cancer cells to PTX or platinum drugs. Thus, the survival rate of patients with ovarian cancer may be improved (Choi et al., 2019). The study of Kim et al. indicated that CMG002 inhibits hepatocellular carcinoma (HCC) tumour growth through *in vivo* and *in vitro* experiments, for the first time (Kim et al., 2019). The team used the MTT assay to process the Huh-7 and HepG2 cell lines and found that CMG002 reduced the viability of the two cell lines in a dose-dependent manner (Baiz et al., 2009; Cervello et al., 2012). Additionally, western blotting showed that CMG002 inhibited the phosphorylation of Akt and S6 in the PI3/Akt/mTOR pathway of the above two cell lines, but neither showed an effect on ERK phosphorylation. The combined use of sorafenib led to decreased phosphorylation of Akt, S6 and ERK in the two cell lines.

## 13 PF-04979064 (24)

To identify alternative candidates to structurally differentiate PF-04691502 (**11**) from the tricyclic imidazo [1,5]naphthyridine series, Compound **11** was developed using a fast follower approach to dactolisib (**7**) and was determined to be an interesting pilot (Cheng et al., 2013).

**FIGURE 6:**
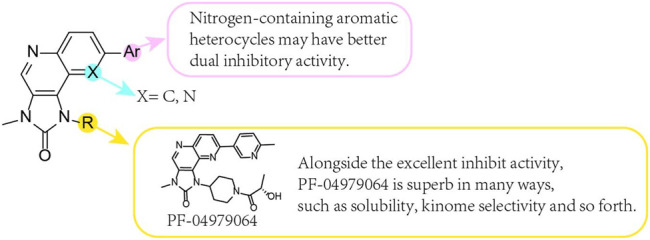
SAR of imidazonaphthyridone core compounds.

It was shown that PF-04979064 (Figure 6), a potent ATP competitive dual PI3K/mTOR inhibitor, is an outstanding *in vitro* inhibitor with excellent solubility, high LipE, excellent kinome selectivity in mice, andin combination with CYP and AO-mediated metabolism an acceptable prediction of human clearance rate afterwards. The PI3Kα Ki and mTOR Ki were 0.130 and 1.42 nM, respectively.

PF-04979064 was determined to be an alternative candidate to PF-04691502 (**11**). It warrants further study in the human body and CT (Yuan et al., 2011; Cheng et al., 2013).

## 14 Small Molecule Macrocyclic Compound: MCX83 (25)

MCXs, a series of small molecule macrocyclic compounds, are novel PI3K/mTOR dual inhibitors designed and synthesized based on omipalisib (**3**) (Álvarez et al., 2021), that show powerful antitumour effects. The macrocyclisation strategy used to regulate the dual inhibition of PI3K/mTOR has strong biochemistry, cell activity and kinase selectivity and has outstanding drug-like properties. Except for compounds with thienopyrimidine and pyrrolopyridine scaffolds, most MCXs showed effective PI3K-α inhibition (IC_50_ < 20 nM). Exploring MCX has revealed that the inhibitory effect on PI3Kα is more pronounced than that on mTOR, although many MCX can be classified as dual PI3K-α/mTOR inhibitors (ratio <10; IC_50_ value in the nanomolar range).

Substituting 1,5-naphthyridine for the quinoline ring provides a highly effective dual inhibitor (82–84). Compared with quinoline analogues, the improvement of mTOR activity in this series was significant. Therefore, compared with analogue 79, the potency of MCX 82 is increased 40 times (IC_50_ = 16.5 and 655 nM). Compared with the benzenesulfonamide analogue 84 (IC_50_ = 3.7 and 23.4 nM), the presence of fluorine atoms in the benzenesulfonamide fragment (83) once again increased the activities of PI3K-α and mTOR (IC_50_ = 0.8 and 3.3 nM). Among them, MCX83 (IC_50_ of PI3K and mTOR: 0.8 and 3.3, respectively; ratio: 4.1) showed significant selectivity to a group of 468, high *in vitro* metabolic stability and good pharmacokinetic parameters and could be taken orally without significant CYP450 and h-ERG binding inhibition. The effect of the thienopyrimidine scaffold is more pronounced in mTOR, producing almost inactive MCX. Compared with quinazoline analogues, the reduction in mTOR activity was significant (85 vs. 119 and 86 vs. 120; IC_50_ = 5,790 nM vs. 30.5 nM, > 10000 vs. 16.7 nM).

Regarding kinase selectivity, although both compounds are highly selective, MCX 83 has a lower number of off-targets than GSK-2126458. This finding may indicate that MCX 83 has better selectivity than the other GSK-2126458. Compared with the benzene sulfonamide analogue MCX 84 (IC_50_ = 3.7 and 23.4 nM), the presence of fluorine atoms in the benzene sulfonamide fragment (MCX 83) once again increased the activities of PI3K-α and mTOR. This profile shows that the compound can be used as a suitable drug candidate for future *in vivo* PK-PD and mouse cancer model efficacy studies.

## 15 Discussion

The phosphatidylinositol 3-kinase (PI3K)-protein kinase B (Akt)-mammalian target of rapamycin (mTOR) transduction pathway plays a key role in various cell functions, including cell growth, proliferation, motility, differentiation, and survival (Guertin and Sabatini, 2007; Dienstmann et al., 2014).

Abnormal activation of the PI3K/Akt/mTOR pathway at various signal levels is often observed in multiple human malignancies, providing a solid preclinical theoretical basis for the use of drugs targeting this pathway. The PI3K/mTOR axis is among the most frequently interrupted intracellular pathways in human cancers. It significantly promotes tumour progression and the development of drug resistance to chemotherapeutics (Lindblad et al., 2016). As mentioned above, among the known PI3K/mTOR inhibitors, some reportedly have an arylsulfonamide scaffold, including omipalisib, SN202, NSC765844 and CMG002, indicating that arylsulfonamide is a potential privileged structure of dual PI3K/mTOR inhibitor (Han et al., 2016). Also, the PI3K inhibitor pictilisib contains this structure, which could serve as a starting point in the process of synthesising more compounds. It is noteworthy that paxalisib and bimiralisib are blood-brain barrier cross-able drugs, among the known dual PI3K/mTOR inhibitors (Heffron et al., 2016; Beaufils et al., 2017).

Many PI3K/mTOR dual inhibitors are now on the market and in use, such as dactolisib, samotolisib, voxtalisib and so forth. New drugs are still being designed and synthesized, and preclinical studies are continuously being performed. Many of these drugs, if not most, have shown good selectivity. The effect of dual PI3K/mTOR inhibitor treatment is reportedly better than that of a single inhibitor (Co iffier and Ribrag, 2009). PI3K/mTOR dual inhibitors are considered to be critical in cancer treatment and have been used in clinical trials. Some inhibitors also demonstrated their role in destroying tumour cells through autophagy. Although the drug resistance of PI3K/mTOR dual inhibitors is generally less than that of single-target inhibitors, the current drug resistance of inhibitors remains a factor affecting long-term treatment (Feldman et al., 2009, 2; Zhang et al., 2011).
